# Supplementary material for: Identification of Freezing-Responsive microRNAs and Their Targets in Chinese Jujube by Small RNA and Degradome Sequencing
Source: Int J Mol Sci. 2026 Sep 4;27(17):7912. doi: 10.3390/ijms27177912 (PMC13566890; doi:10.3390/ijms27177912)
Supplement: Supplementary file 1 [file ijms-27-07912-s001.zip › Supplementary Material S2/novel_miR_102.pdf]

Provisional ID	:	novel_miR_102
Score total	:	0.3
Score for star read(s)	:	-1.3
Score for read counts	:	0
Score for mfe	:	0
Score for randfold	:	1.6
Score for cons. seed	:	
Total read count	:	53
Mature read count	:	53
Loop read count	:	0
Star read count	:	0

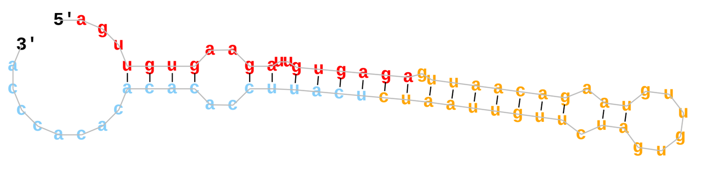

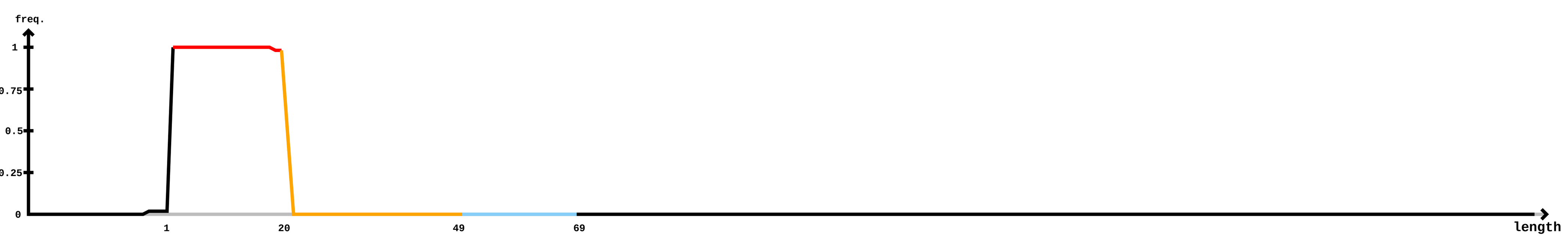

Mature Star

```
5'- uacacguaccguguccauuagaaguugugaagauugugagaguuaacagaauguugugaucuuguuaaaucucauuccacacacacacccacaccccgcggggcgnnnnnnnnnnnnnnnnnnnnnnnnnnnnnnnnnnnnnnnnnnnnnnnnnnnnnnnnnnnnnnnnnnnnnnnnnnnnnnnnnnnnnnnnnnnnnnnnnnnnnnnnnnnnnaagguuuugaaaaccaaccaaaaccgaguauuggucg -3'   exp
    .(((((...)))))(((.((...((((((......((((((.(((((((.((......)).)))))))))))))......)))).))...(.(((....))).)...................................................................................................................(((((((.........)))))))...)).)))...   reads   mm   sample
    ..................uagaaguugugaagauuguga.......................................................................................................................................................................................................................   1   0   JCK-2
    ......................aguugugaagauugugaga.....................................................................................................................................................................................................................   27   0   LCK-2
    ......................aguugugaagauugugaga.....................................................................................................................................................................................................................   26   0   LCK-1
```
